# Occurrence and Determination of *Alternaria* Mycotoxins Alternariol, Alternariol Monomethyl Ether, and Tentoxin in Wheat Grains by QuEChERS Method

**DOI:** 10.3390/toxins14110791

**Published:** 2022-11-12

**Authors:** Nikola Puvača, Giuseppina Avantaggiato, Jordan Merkuri, Gorica Vuković, Vojislava Bursić, Magdalena Cara

**Affiliations:** 1 Department of Engineering Management in Biotechnology, Faculty of Economics and Engineering Management in Novi Sad, University Business Academy in Novi Sad, Cvećarska 2, 21000 Novi Sad, Serbia; 2 Institute of Sciences of Food Production (ISPA), National Research Council (CNR), Via Amendola, 70126 Bari, Italy; 3 Department of Plant Protection, Faculty of Agriculture and Environment, Agricultural University of Tirana, Koder Kamez, 1029 Tirana, Albania; 4 Faculty of Agriculture, University of Belgrade, Nemanjina 6, 11080 Belgrade-Zemun, Serbia; 5 Department for Phytomedicine and Environmental Protection, Faculty of Agriculture, University of Novi Sad, Trg Dositeja Obradovića 8, 21000 Novi Sad, Serbia

**Keywords:** fungi, emerging mycotoxin, toxicity, grains, exposure, QuEChERS, *Alternaria*

## Abstract

The *Alternaria* mycotoxins such as alternariol (AOH), alternariol monomethyl ether (AME), and tentoxin (TEN) are mycotoxins, which can contaminate cereal-based raw materials. Today, wheat is one of the most important crops in temperate zones, and it is in increasing demand in the Western Balkans countries that are urbanizing and industrializing. This research aimed to investigate the occurrence and determine the concentration of *Alternaria* mycotoxins AOH, AME, and TEN in wheat samples from the Republic of Serbia and the Republic of Albania, harvested in the year 2020 in the period between 15 June and 15 July. A total of 80 wheat grain samples, 40 from each country, were analyzed by an QuEChERS (quick, easy, cheap, effective, rugged, and safe) method. From the obtained results, it can be seen that the mean concentration of AOH was 3.3 µg/kg and AME was 2.2 µg/kg in wheat samples from Serbia, while TEN from both Serbia and Albania was under the limit of quantification (<LOQ). The maximum of AOH and AME mycotoxins was recorded only in wheat grain samples collected in the Republic of Serbia (5.3 and 2.3 µg/kg). In conclusion, *Alternaria* mycotoxins have concentrations above the LOQ, which could be potentially considered a health hazard to both humans and animals.

## 1. Introduction

Wheat has a great deal of economic importance as well as its contribution to the diets of humans [1,2,3] and food animals [4], which cannot be ignored. According to official FAOstat data [5], in the year 2020, recorded wheat production in the Republic of Serbia and Republic of Albania was 2.8 million and 0.2 million metric tons, respectively. This makes wheat one of the most important crops in terms of these Western Balkans countries’ annual production. As a result, wheat is becoming increasingly popular in markets outside its climatic region [6,7,8,9,10]. Industrialization and westernization have resulted in a growing demand for unique food products made from wheat and wheat flour [11,12,13,14,15], which could be easily contaminated with mycotoxins [16]. A variety of functional ingredients can be produced from wheat [17] because of the unique properties of the gluten protein fraction [18]. Traditional foods are more difficult to prepare, and western lifestyles may call for more convenient products [19,20].

**Figure 1:**
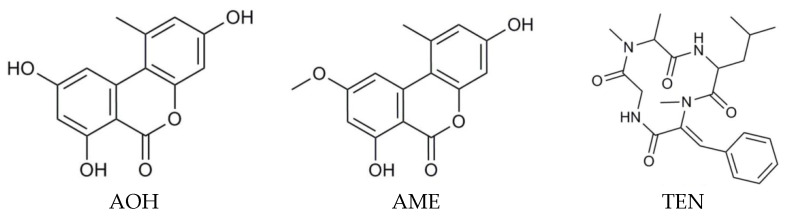
Structure of the *Alternaria* mycotoxins AOH, AME, and TEN [37]. AOH—alternariol; AME—alternariol monomethyl ether; TEN—tentoxin.

The *Alternaria* mycotoxins such as alternariol (AOH), alternariol monomethyl ether (AME), and tentoxin (TEN) (Figure 1) are mycotoxins, which can contaminate cereal-based raw materials [21,22,23,24,25]. It has been reported that *Alternaria* fungi produce a total of at least 70 different mycotoxins [26,27,28]. Most *Alternaria* mycotoxins in grains have contamination levels below 100 µg/kg and maximum concentration levels below 1000 µg/kg [29]. In addition, the seriousness of mycotoxins in the food chain such as milk [30], milk products [31,32], nuts [33,34], and other agricultural products [35,36] must not be forgotten.

**Figure 2:**
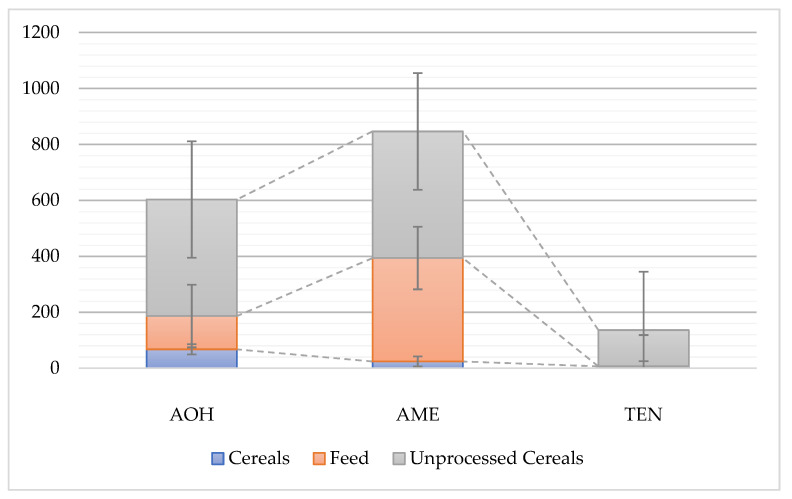
The average values (µg/kg) of worldwide concentrations of detected *Alternaria* mycotoxins AOH, AME, and TEN in food and feed commodities, [46]. AOH—alternariol; AME—alternariol monomethyl ether; TEN—tentoxin.

Since *Alternaria* species are highly adaptable to environmental conditions, including their ability to grow and produce toxic secondary metabolites at low temperatures [38], they can infect every stage of the food chain [39]. *Alternaria* mycotoxins are prevalent in a wide range of food and feed commodities [40], from cereals [41,42], fruits [43], and vegetables to drinks such as juices [44] and wines [45] (Figure 2). Due to this, humans are easily exposed to *Alternaria* mycotoxins through the environment and contaminated foods.

Other plant pathogens can live as endophytes on plant tissues asymptomatically, but *Alternaria* species generally cause plant diseases. Humans and animals are believed to be affected by AOH and AME, which are genotoxic, mutagenic, and carcinogenic [47], while plants are affected by TEN, which inhibits chloroplast production and causes chlorosis [48]. As a result of AOH interaction with deoxyribonucleic acid (DNA) topoisomerase, reactive oxygen species (ROS) are formed, and DNA strands can be broken into single and double strands. A decrease in cell proliferation is caused by the arrest of the cell cycle in the G2-phase checkpoint, which may be caused by the attempt to repair DNA damage. Furthermore, AOH inhibits macrophage differentiation in THP-1 monocytes and decreases TNF-α secretion [49]. It interacts with steroidogenesis and exhibits an estrogenic response similar to estradiol. In addition, AME and AOH decreased progesterone formation by decreasing the abundance of a key enzyme [50]. Recent studies have shown that the European population has exceeded the threshold of toxicological concern (TTC) for AOH and AME by consuming bakery products, juices, and tomato products [46,51,52,53]. An in-depth analysis involving cereals-based products, and juices, resulted in a 95th percentile dietary exposure exceeding the TTC for TEN, AOH, and AME, with a factor of 1.4, 12, and 60, respectively [54].

Detecting *Alternaria* mycotoxins in food and feed products contaminated by it is a vital part of food and feed safety assessment [55,56]. Mycotoxins from *Alternaria* can be measured instrumentally using the following methods: enzyme-linked immunosorbent assay (ELISA) [57], thin layer chromatography (TLC) [37], gas chromatography (GC) [58] coupled to a mass spectrometry (MS) detector, liquid chromatography (LC) [59] coupled to an ultraviolet detector (UV), and mass spectrometry detector (LC-MS), or a diode array detector (DAD) [60]. On the other hand, the GC and GC tandem MS rarely detect *Alternaria* mycotoxins because they are stable and nonvolatile. Solid phase extraction (SPE) or QuEChERS extraction is often necessary to achieve satisfactory sensitivity due to the complexity of investigated food and feed matrices [61]. The QuEChERS is a quick, easy, cheap, effective, rugged, and safe sample pretreatment technology based on dispersive SPE and has been successfully used in detecting *Alternaria* mycotoxins [62]. Several mycotoxins found in food and feed samples have already been analyzed using the QuEChERS approach [63,64,65,66].

Having in mind that there are remaining knowledge gaps regarding the studied emerging *Alternaria* mycotoxins in two important key factors for a proper risk assessment, including occurrence, and toxicity data, this research aimed to investigate the occurrence and determine the concentration of *Alternaria* mycotoxins AOH, AME, and TEN in wheat samples from the Republic of Serbia and Republic of Albania harvested in the year 2020.

## 2. Results

The *Alternaria* mycotoxins AOH, AME, and TEN were quantified using a validated approach to ensure accuracy and reliability following the Commission Regulation (EC) No 401/2006 [67] and Commission Recommendation EU/2022/553 [68]. The procedural standard calibration demonstrated good linearity in the concentration range of 2–40 µg/kg for all the investigated mycotoxins with a coefficient of linearity (R^2^) of >0.99.

The limit of detection (LOD) is the lowest concentration of a substance that is detectable by a given measurement procedure and it was calculated by MassHunter software (Santa Clara, CA, USA) (signal/noice = 5). The limit of quantification (LOQ) is the lowest spike level (2 µg/kg) of the validation to fulfil the method’s performance acceptability criteria. The obtained average recovery values after spiking blank wheat samples at three levels is 2, 4, and 10 µg/kg, with the relative standard deviation (%RSDr) for the repeatability shown in Table 1.

Table 2 represents the results of our investigation of the occurrence and concentration of *Alternaria* mycotoxins AOH, AME, and TEN in wheat samples from the Republic of Serbia and the Republic of Albania. From the presented results it can be seen that the mean recorded concentration of AOH was 3.3 ± 1.3 µg/kg, AME was 2.2 ± 0.1 µg/kg in the samples of wheat collected in the Republic of Serbia. The same table shows that in samples collected from the Republic of Albania, concentrations of AOH, AME, and TEN was under the LOQ, respectively. The same tendency regarding the concentration of TEN in wheat samples from the Republic of Serbia were recoded.

The highest recorded concentration of AOH and AME mycotoxins was in wheat grain samples from the Republic of Serbia (5.3 and 2.3 µg/kg).

From Table 2, it can be seen that the median trend for the concentration of *Alternaria* mycotoxins in wheat samples collected from the Republic of Serbia is TEN < AME < AOH.

These results show that the highest percentage of investigated wheat grain samples are contaminated with AOH mycotoxins, followed by AME in the samples from the Republic of Serbia, whereas the contamination of AOH, AME, and TEN mycotoxins in wheat collected from the Republic of Albania was under the LOQ.

## 3. Discussion

A hexaploid species called “common” or “bread” wheat is the most common wheat species grown worldwide [69]. Globally, wheat, a tetraploid species (*Triticum durum*) that thrives in hot, dry climates around the Mediterranean Sea and similar climates elsewhere, is produced in quantities of 35–40 million tons.. There are about 150 million tons of wheat traded annually, making it a global commodity [70]. Wheat consumption has been found to increase with urbanization and industrialization in countries that have adopted a “western lifestyle” [1]. A wide variety of food and feed crops such as wheat, corn, or cereals are contaminated with *Alternaria* fungi that produce mycotoxins such as AOH, AME, tenuazonic acid (TeA), and TEN, which are the most significant [71,72,73]. Our investigation has focused on the determination of *Alternaria* mycotoxins AOH, AME, and TEN in wheat samples from the Republic of Serbia and the Republic of Albania.

Romero Bernal et al. [74] have used an HPLC-DAD methodology to determine the concentration of AOH and AME mycotoxins in wheat grain, bran, and flour samples. The LOD in their investigation was 3.4 and 4.5 µg/kg for AOH, and AME, respectively. In comparison to our investigation, our LOD for AOH, AME, and TEN was 0.5, 0.3, and 0.5 µg/kg, respectively. The concentrations of investigated mycotoxins in Romero Bernal et al. [74] samples were 3.1, 4.5, and 12 µg/kg for AOH, AME, and TeA, respectively. Our investigation has recorded lower concentrations of AOH, AME, and TEN. Mycotoxin inspection has recorded a wide range of AOH (5–72 µg/kg), AME (5 µg/kg), TEN (5–27 µg/kg), citreoviridin (10–57 µg/kg), and mycophenolic acid (10–95 µg/kg), in cereals produced in different regions of Russia [75]. In comparison to Russia, our results of investigated wheat grains from the Republic of Serbia and the Republic of Albania have significantly lower concentrations of these mycotoxins. Furthermore, investigations by Topi et al. [53] conducted in the Republic of Albania from 2014 to 2015 have shown higher concentrations of AOH, AME, TEN, and TeA detected by an LC-MS/MS method. In their investigation, the highest concentration of total mycotoxins in corn was 1283 μg/kg, while the maximum concentration in wheat was 175.7 μg/kg, and the major recorded mycotoxin was TeA. In our investigation, the concentrations of AOH, and AME, and TEN mycotoxins in samples from the Republic of Albania, analysed by the QuEChERS method were under the LOQ. Additionally, Vuković et al. [76] suggest a “dilute-and-shoot” method for the *Alternaria* mycotoxins determination in wheat grains as a simple method with easy sample preparation, which has good accuracy and precision.

Argentina’s major producing region has been found to have *Alternaria* mycotoxins naturally occurring in malting barley grains. Castañares et al. [77] conducted the study intending to analyze the occurrence of AOH, AME, and TeA in malting barley grains. As in our research, with samples from Serbia and Albania where the most dominant mycotoxin was AOH, in Argentina, the situation was the same with the most frequent mycotoxin, AOH, in the concentration of 712 µg/kg. The same authors have found a negative correlation between environmental temperature and AOH mycotoxin concentration [77].

On the other hand, Gashgari et al. [78] investigated the toxicity of different *Alternaria* strains in a bioassay with a model bacteria, *Bacillus subtilis*; they found that all investigated strains are producing the toxins. Furthermore, they have concluded that the occurrence of mycotoxins has not always been associated with fungal toxicity.

Molecular identification and mycotoxin production by *Alternaria* on Durum wheat was conducted by Masiello et al. [79]. The authors have shown that 84 strains, phylogenetically grouped in the *Alternaria* section, produced AOH, AME, and TeA with values of 8064, 14,341, and 3683 µg/g, respectively [79]. Schiro et al. [80] investigated the differences in distribution and spore deposition of *Alternaria* and *Fusarium* fungi. Based on the obtained results it appears that the two fungi have different patterns of spore distribution and deposition, while the abundances were assessed genetically using qPCR-based techniques [80].

In research by Kifer et al. [81] in the neighboring country Croatia, the seven *Alternaria* mycotoxins metabolites were detected in cereals collected from two locations. The median values in Croatian samples ranged from 0.6–0.7 µg/kg (AME), 5.1–6.4 µg/kg (AOH), and 2.4–4.0 µg/kg (TEN). A similar tendency was observed in our obtained results regarding the AOH mycotoxins concentrations. A study in experimental animals found that TeA was the most toxic of the *Alternaria* metabolites, leading to an increased feed conversion ratio, losses in body weight, and the occurrence of lesions in the digestive tract [82]. In other research with pigs, AME, and TEN mycotoxins did not cause significant cytotoxicity in animals’ jejunal epithelial cells, while TeA had an IC_50_ 100 times greater than the median concentration detected in feed [83]. In their dietary experiment with broiler chickens, Puvača et al. [84] showed that the wheat contaminated with *Alternaria* mycotoxins in broilers’ nutrition negatively affects growth, decreases oxidative protection, and exhibits a negative influence on overall chicken welfare.

Nevertheless, according to the European Food Safety Authority (EFSA), *Alternaria* mycotoxins metabolites have been detected in feedstuffs, and the effects of these metabolites on animals have not been sufficiently assessed [85]. Therefore, further investigations on the negative effects of *Alternaria* mycotoxins contaminated food and feed are necessary.

## 4. Conclusions

Based on the obtained results it can be seen that the average concentration of AOH was 3.3 µg/kg, AME was 2.2 µg/kg, and TEN was under the LOQ, regarding the samples collected in the Republic of Serbia. Our results have shown that concentrations of all three investigated *Alternaria* mycotoxins collected in the Republic of Albania was under the LOQ. The maximal concentration of AOH and AME mycotoxins was recorded in wheat grain samples from the Republic of Serbia (5.3 and 2.3 µg/kg, respectively).

Furthermore, using the results obtained in our investigation, food and feed safety authorities could determine the need for their regulation based on the risk assessment of exposure to *Alternaria* mycotoxins. Food and feed supply chains are challenged by the high variation in the amounts of toxins produced by different *Alternaria* species and strains. Even though the QuEChERS method allows for the detection and quantification of a wide variety of fungal metabolites in cereals, the toxicological significance of the data obtained needs further investigation.

In conclusion, *Alternaria* mycotoxins in our research have recorded concentrations above the LOQ, which could be a potential health hazard to both humans and animals.

## 5. Materials and Methods

### 5.1. Chemicals and Reagents

The analytical standards of the AOH, AME, and TEN were purchased from Sigma-Aldrich (Zwijndrecht, The Netherlands). The standards were dissolved with 1.00 mL of methanol (MeOH) to obtain 0.1 mg/mL stock solutions. All stock solutions were kept at 4 °C. The mixtures of all the *Alternaria* mycotoxins (working standards) were prepared in acetonitrile (MeCN) in the final concentrations of 10 and 1 µg/mL. These solutions were used for spiking the blank samples for the calibration and recovery analyses. The MeOH and MeCN were HPLC ultra-gradient grade obtained from Sigma-Aldrich (Zwijndrecht, The Netherlands). The ammonium formate was analytical grade purchased from Merck (Darmstadt, Germany). The products Dispersive SPE 15 mL, fatty samples (EN) (part no.5982-51565), and QuEChERS extraction kit original method (part no. 5982-7550) were purchased from Agilent Technologies (Santa Clara, CA, United States).

### 5.2. Sample Collection, Spiking, and Extraction

A total of 80 samples of wheat grains, 40 from each country, were analyzed for the presence of *Alternaria* mycotoxins, i.e., AOH, AME, and TEN. Wheat grain samples (*Triticum aestivum*) were collected in post-harvest time in the season of 2020 from the region of Serbia (Vojvodina) and Albania (Durrës). Obtained samples were collected with the appropriate equipment, such as a probe for stationary grain and a diverter-type mechanical sampler, using a sampling pattern and procedures designed to collect samples from all areas of the lot. The appropriate size of wheat grain sample, between 1.5 and 2.5 kg, was taken from a truck with adequately identifiable and labeled bags. Collected samples were handled in such a way as to maintain representativeness. Samples were stored in a cool and dry place in triple-lined paper breathable bags to avoid mold growth and an increase in the sample moisture level over 14%. The sampling was performed following the Commission Regulation (EC) No 401/2006 [67]. The collected samples were ground into a fine powder before the analysis. The fine powder of wheat grains was achieved by milling the samples on an MLU-202 automatic laboratory mill (Bühler, Wuxi, China), with the flour extraction rate at around 70%.

**Figure 3:**
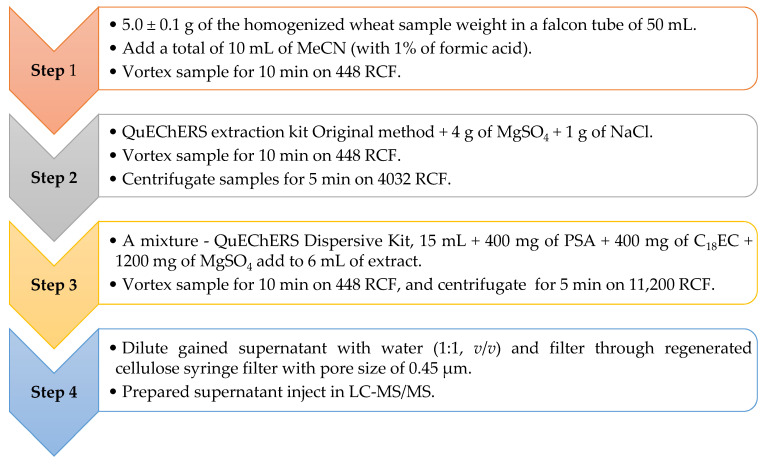
The steps of the AOH, AME, and TEN extractions. AOH—alternariol; AME—alternariol monomethyl ether; TEN—tentoxin.

The *Alternaria* mycotoxins were extracted from ground wheat powder samples using the QuEChERS method described in Figure 3.

### 5.3. Instrumentation

The HPLC Agilent 1290 Infinity II chromatograph equipped with a quaternary pump, multi sampler, and column compartment thermostat was used for the *Alternaria* mycotoxins detection. The HPLC system was coupled to an Agilent 6470B LC/TQ triple quadrupole mass spectrometer with AJS ESI (Jet Stream Technology Ion Source). An Agilent Zorbax Eclipse Plus C18 column was used for the chromatographic separation. The column temperature was held at 35 °C and the injection volume for the LC system was 2 µL. The chromatographic separation of the AOH, AME, and TEN were carried out with a mobile phase consisting of water (A) and acetonitrile (B), both containing 10 mM ammonium formate, in a gradient mode and flow rate of 0.3 mL/min. A gradient elution started at 5% of B and held for 1 min. This composition was increased to 40% B at 7 min, 90% B at 8 min, and then held for 2 min. The composition of the mobile phase returned to the initial conditions in 1 min and the system was equilibrated for 2 min. The total running time was 11 min. The ESI source was used with the following settings: drying gas (nitrogen) temperature of 200 °C, drying gas flow rate 16 L/min, nebulizer pressure 30 psi, sheath gas temperature of 300 °C, sheath gas flow 12 L/min, and capillary voltage 3000 V. The detection was performed using the dynamic multiple reactions monitoring mode (dMRM). The Agilent MassHunter software (v. B.10.0 SR1 Agilent Technologies, 2006–2019, Santa Clara, CA, USA) was used for the optimization and quantification.

## Figures and Tables

**Table 1:** Validation parameters of AOH, AME, and TEN in wheat.

Mycotoxins	Rt, min	LOD, µg/kg	LOQ, µg/kg	R^2^	Recovery, % (%RSDr)
AOH	6.25	0.5	2.0	0.9998	107.6 ± 6.5
AME	7.93	0.3	2.0	0.9998	108.0 ± 6.5
TEN	6.26	0.5	2.0	0.9915	110.1 ± 6.5

AOH—alternariol; AME—alternariol monomethyl ether; TEN—tentoxin; Rt—retention time; LOD—limit of detection; LOQ—limit of quantification; R^2^—coefficient of linearity; %RSDr—relative standard deviation.

**Table 2:** Occurrence of AOH, AME, and TEN in the investigated wheat grain samples.

	AOH		AME		TEN	
Sample Origin	Serbia	Albania	Serbia	Albania	Serbia	Albania
Mean (μg/kg) ± SD	3.3 ± 1.3	-	2.2 ± 0.1	-	-	-
Minimal concentration (μg/kg)	2.1	-	2.2	-	-	-
Maximal concentration (μg/kg)	5.3	<LOQ	2.3	<LOQ	<LOQ	<LOQ
Number of positive samples	4	0	2	0	0	0
Pooled SE	0.2	-	0. 0	-	-	-

AOH—alternariol; AME—alternariol monomethyl ether; TEN—tentoxin; SD—standard deviation; Pooled SE—standard error; <LOQ—below the limit of quantification.

## Data Availability

Data are contained within the article.
